# Transcriptional mutagenesis dramatically alters genome-wide p53 transactivation landscape

**DOI:** 10.1038/s41598-020-70412-4

**Published:** 2020-08-11

**Authors:** Shuo Liang, Monika Ezerskyte, Jingwen Wang, Vicent Pelechano, Kristian Dreij

**Affiliations:** 1 Unit of Biochemical Toxicology, Institute of Environmental Medicine, Karolinska Institutet, 171 77 Stockholm, Sweden; 2 Science for Life Laboratory, Department of Microbiology, Tumor and Cell Biology, Karolinska Institutet, 171 77 Stockholm, Sweden

**Keywords:** Cell biology, Tumour-suppressor proteins

## Abstract

The transcriptional error rate can be significantly increased by the presence of DNA lesions that instruct mis-insertion during transcription; a process referred to as transcriptional mutagenesis (TM) that can result in altered protein function. Herein, we determined the effect of *O*^6^-methylguanine (*O*^6^-meG) on transcription and subsequent transactivation activity of p53 in human lung H1299 cells. Levels of TM and effects on transactivation were determined genome wide by RNA-seq. Results showed that 47% of all p53 transcripts contained an uridine misincorporation opposite the lesion at 6 h post transfection, which was decreased to 18% at 24 h. TM at these levels reduced DNA binding activity of p53 to 21% and 80% compared to wild type p53, respectively. Gene expression data were analysed to identify differentially expressed genes due to TM of p53. We show a temporal repression of transactivation of > 100 high confidence p53 target genes including regulators of the cell cycle, DNA damage response and apoptosis. In addition, TM repressed the transcriptional downregulation by p53 of several negative regulators of proliferation and differentiation. Our work demonstrates that TM, even when restricting its effect to an individual transcription factor, has the potential to alter gene expression programs and diversify cellular phenotypes.

## Introduction

Various DNA lesions from plethora of agents present in our environment pose a constant threat to cells’ genomic integrity, regardless of their proliferation state. Mutagenic potential and consequences of DNA lesions during DNA replication are well established and has been linked to genomic instability and a variety of diseases, including cancer^1^. However, majority of mammalian cells exist in non-proliferative or slow-growing state in their native tissue and their homeostasis and viability largely depend on transcription^2^. Encounters of elongating transcription machineries with DNA lesions can affect RNA synthesis by not only causing transcriptional arrest, which in turn activates transcription-coupled repair^3,4^, but also allowing bypass with potential misincorporation event into nascent RNA resulting in mutant transcript through a process known as transcriptional mutagenesis (TM)^2^.

Frequently occurring small lesions formed by alkylation, oxidation and deamination of the bases in DNA has been shown to induce events of TM in mammalian in vitro^5–7^ and in vivo systems^8–12^ resulting in production of mutated transcripts and proteins with altered functions. The role of these seemingly transient changes of the cellular phenotype in disease development remains unclear. However, increasing lines of evidence suggest that TM could be a mechanism which contributes to tumorigenesis by inactivation of tumour suppressors and/or activation of oncogenes^10,12,13^.

Inactivation of tumour suppressor genes that negatively regulate cell proliferation is one of the key hallmarks of tumorigenesis^1^. *TP53* is one of the most important canonical tumour suppressor genes in humans. The p53 protein primarily functions as a transcription factor and exerts its tumour suppressive function mainly through transcriptional activation of a large number of target genes, which in turn mediate multiple important cellular processes including regulation of cell cycle, apoptosis, DNA repair, metabolism, autophagy, translational control, and feedback mechanisms^14^. This is managed by a differential time-dependent transactivation of target genes with cell-cycle genes generally being induced early upon p53 activation while induction of most apoptotic genes is a relatively later event^15,16^. Although p53 is a transcriptional activator, it can also mediate downregulation indirectly via *CDKN1A*/p21 and mainly of cell cycle genes^17^.

Not surprisingly, the loss of p53 function by mutation has been found in more than half of all human cancers and, unlike other tumour suppressors, the vast majority of *TP53* mutations are missense mutations occurring within the DNA-binding domain of the protein resulting in a dominant-negative phenotype with a diminished ability to transactivate target genes e.g. the hot spot mutation R248W^18^. Also, p53 has been shown to acquire gain-of-function mutations with new additional oncogenic functions, although this is not fully established^19,20^. We recently showed that *O*^6^-meG-induced TM at codon 248 reduced the p53 tumour-suppressor function through deregulation of cell cycle control and impaired activation of apoptosis in human cells^10^. This was partly mediated through deregulated transactivation of some core p53 target genes including *CDKN1A* and *BBC3*. The genome-wide impact of TM on p53 function as transcription factor is however not known. Here, the time-dependent impact of TM at codon 248 on the transcriptional regulation of p53 target genes was assessed on a genome-wide level in human cells by RNA-seq.

## Results

### Induction of TM in p53 by O^6^-meG reduces p53 DNA binding capacity

The extent of TM in p53 transcripts at the position corresponding to codon 248 was determined by RNA-seq. To determine the time-dependence of p53 TM, this was measured at an early (6 h) and late (24 h) time point post transfection. As expected, cells expressing wild type or the RW mutant p53 protein displayed respectively < 1% and > 99% of uridine incorporation in transcripts at this position (Table 1). Transcription past *O*^6^-meG in cells with active *O*^6^-alkylguanine-DNA alkyltransferase (AGT) resulted in low levels of uridine incorporation, 0.24% and 0.39% at the two time points, respectively. Inactivation of AGT by *O*^6^-benzylguanine (*O*^6^-bzG)^10^ increased the frequency of uridine incorporation opposite the lesion by two orders of magnitude to 47.3% (*P* < 0.0001) and 18.0% (*P* < 0.0005) TM at the early and late time points, respectively. No other misincorporations were detected. We have previously shown that the pre-treatment with *O*^6^-bzG is non-cytotoxic and inhibits the AGT activity > 95% for up to 24 h^10^. The level of TM at the early time point was further confirmed by a PCR-based approach which showed 51.0 ± 3.0% (mean ± SE, *n* = 4) uridine incorporation opposite the lesion in cells with inactive AGT.

**Table 1:** Analysis of uridine incorporation at codon 248 and the position corresponding to the site-specific *O*^6^-meG. No other misincorporations were detected.

Plasmid	RNA-seq read coverage		U incorporation at codon 248 (%)	
	6 h	24 h	6 h	24 h
pP53^WT^	753	759	0.13 ± 0.13^1^	0
pP53^RW^	799	738	99.9 ± 0.13**	99.7 ± 0.28**
pP53^O6-meG^	819	768	0.24 ± 0.12	0.39 ± 0.22
pP53^O6-meG^ + *O*^6^-bzG	783	737	47.3 ± 4.8**^,††,‡‡^	18.0 ± 3.8*^,†^

^1^Mean ± SE, n = 3.

**P < 0.0001 compared to pP53^WT^, *P < 0.0005 compared to pP53^WT^.

^††^*P* < 0.0001 compared to pP53^O6-meG^, ^†^*P* < 0.0005 compared to pP53^O6-meG^.

^‡‡^*P* < 0.0001 compared to 24 h (one-way Anova).

**Figure 1:**
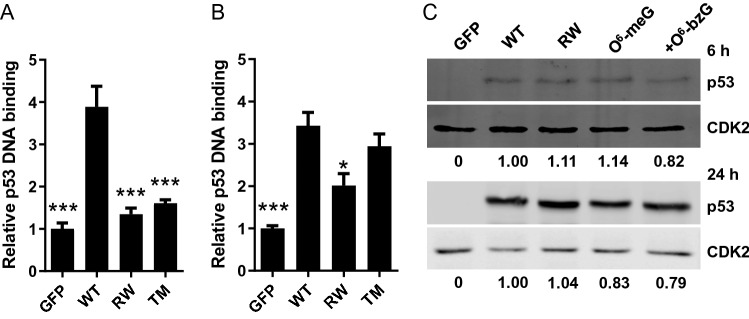
Impact of TM on p53 DNA binding activity. Relative DNA binding activity of p53 at 6 h (**A**) and 24 h (**B**) after transfection. Data show the mean ± SE (*n* = 3) normalized to the activity of GFP expressing cells. *P < 0.05, ***P < 0.001 by one-way ANOVA compared to p53 wild type (WT). TM is the activity in cells transfected with pP53^O6-meG^ with inactive AGT. The positive control caused a 2.8-fold increase of DNA binding activity (not shown). (**C**) Representative blots of p53 protein at 6 h (top) and 24 h (bottom) post transfection by western blot. Relative p53 protein expression levels are shown below the blots. CDK2 was used as a loading control.

The impact of TM on protein function of p53 was assessed by an ELISA-based DNA binding activity assay. The results showed a clear reverse correlation between level of TM and protein activity. At the early time point, TM (*O*^6^-meG with inactive AGT) significantly reduced the DNA binding of p53 to 21% of that of wild type p53 (*P* < 0.001) (Fig. 1A). In agreement with the lower frequency of TM at the later time point, the activity was only slightly reduced to 80% of wild type p53 (Fig. 1B). As expected, the activity of RW mutant p53 was significantly reduced at both time points (*P* < 0.001 and 0.05, respectively). The protein expression level of p53 was similar from the different vectors (Fig. 1C), thereby limiting that any effects on DNA binding activity were due to differences in p53 expression level. A comparison with previous studies also shows that the ectopic expression level of p53 protein seen here are comparable to expression levels of activated p53 in response to DNA damage in human cells^21,22^.

### RNA-seq identifies p53 status-dependent gene expression changes

**Figure 2:**
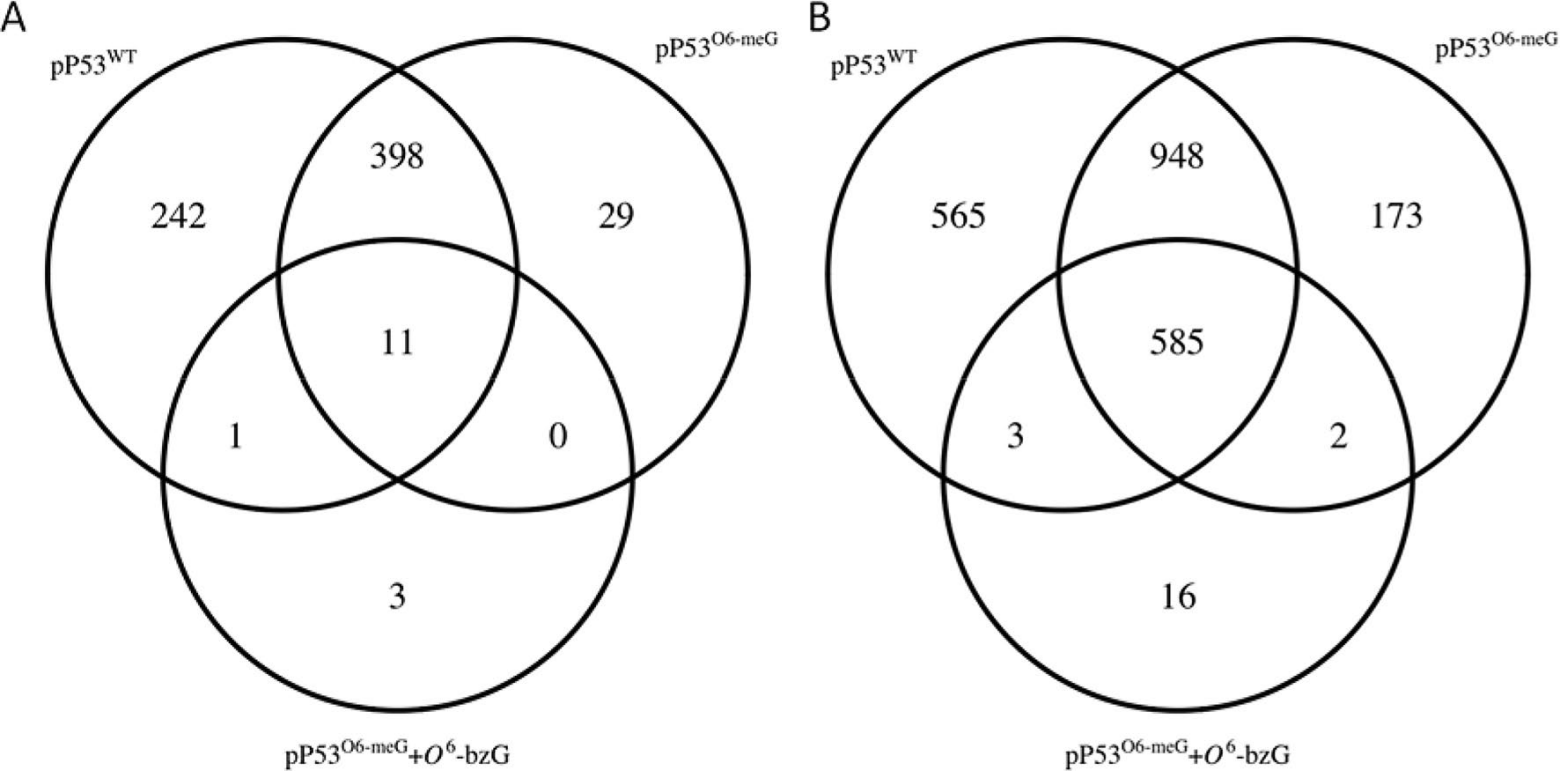
RNA-seq analysis of p53 status dependent differential gene expression. Venn diagrams presenting the DEGs at 6 h (**A**) and 24 h (**B**) post transfection with the indicated vectors. Expression of RW mutant p53 protein did not result in any DEGs except the vector expressed *TP53* (not shown).

Differential gene expression was determined as described in material and methods. A significant change in gene expression was defined as ≥ twofold change compared to the control vector pGFP and with FDR adjusted *P*-value at < 0.05. At 6 h post transfection the total number of differentially expressed genes (DEGs) was 652 (643 up and 9 down) in response to wild type p53, 1 (*TP53*) to RW mutant p53, 438 (407 up and 31 down) to p53 *O*^6^-meG and 15 (14 up and 1 down) to p53 TM (*O*^6^-meG with inactive AGT). At 24 h post transfection the total number of DEGs was 2,101 (1718 up and 383 down) in response to wild type p53, 1 (*TP53*) to RW mutant p53, 1708 (1,354 up and 354 down) to p53 *O*^6^-meG and 606 (577 up and 29 down) to p53 TM. This shows that the number of DEGs increased with time in response to all vectors, except for the RW mutant p53 which did not cause any DEGs (not including the vector expressed *TP53*) compared to pGFP. The overlap between genes with significantly altered expression for cells expressing the *O*^6^-meG-modified p53 vs. wild type p53 was high; ≥ 80% in absence or presence of TM and at both time points (Fig. 2). However, this does not exclude that different pathways or functional categories were affected or affected to a varying degree as a result of TM.

**Figure 3:**
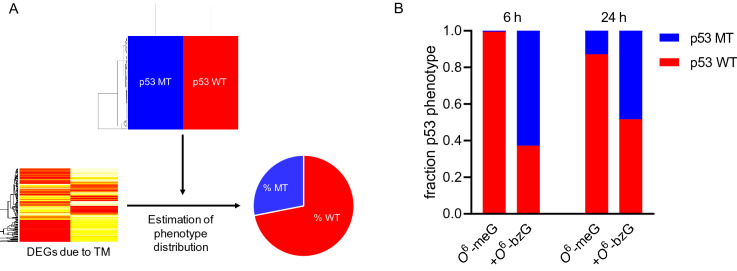
Analysis of relative amount of wild type and mutant p53 expression based on differential expressed genes. (**A**) Schematic of CIBERSORTx approach. Gene expression information after TM was deconvoluted using p53 wild type (WT) and mutant (MT) expressing cells as reference. This allows for using RNA-seq data to estimate the percentage of cells displaying mutant or wild type phenotypes. (**B**) Results are shown for 6 h and 24 h post transfection. Data show the mean ± SE (n = 3). See also Supplementary Table S4.

This was further supported by analysing the DEGs at the two time points using CIBERSORTx, which suggested that TM of p53 affected the phenotype of the cell population considerably (Fig. 3 and Supplementary Table S4). At the early time point, the 47% of mutated p53 transcripts corresponded to a cell population where 65% of the cells displayed a “mutant p53” gene expression program. At the later time point, with lower levels of TM at 18%, this corresponded to a population of cells with 48% displaying a mutated p53 phenotype. These numbers are in good agreement with the DNA binding activity. In accordance with the background levels of TM induced by *O*^6^-meG in presence of active DNA repair, the population corresponded to ≥ 90% wild type p53 at both time points. To further study the cellular effects of p53 TM, more detailed gene ontology and pathway analyses were performed.

### TM reduces the transactivation of p53 target genes which impacts several canonical pathways

**Figure 4:**
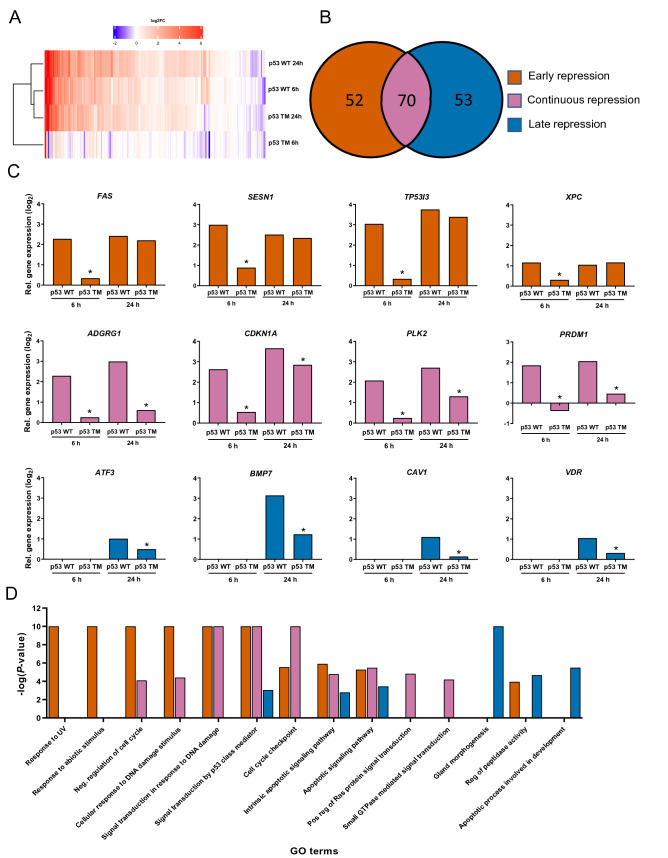
Time-dependent impact of p53 TM on transactivation of high-confidence target genes. (**A**) Heatmap of expression levels of the 343 HCGs in response to WT and TM of p53 at 6 and 24 h. (**B**) Venn diagram presenting three clusters of genes with early (red), continuous (purple) or late (blue) repression of transactivation as a result of p53 TM. (**C**) Examples of genes belonging to the different temporal clusters. Bars are showing relative levels of induction in response to p53 WT and the resulting repression in response to p53 TM, in comparison to pGFP. *P < 0.05 compared to p53 WT. (**D**) Over-represented GO terms in each temporal cluster with *P* < 0.05.

We have previously shown that TM at codon 248 of p53 represses the transactivation of several target genes^10^. To investigate this on a more genome wide level, the transactivation level of 343 high-confidence p53 target genes (HCGs)^14^ was assessed at the same early and late time points as above. In agreement with protein expression level, the gene expression level of *TP53* from the different vectors was similar across all conditions (6.6 ± 0.1 log_2_-fold, mean ± SE, *n* = 8), thereby limiting that any effects on gene expression levels were due to differences in *TP53* expression level. The RNA-seq results showed that expression of wild type p53 induced the expression of 125 and 192 of the HCGs in H1299 cells at 6 and 24 h, respectively (≥ twofold change, *P* < 0.05, Fig. 4A, Supplementary Table S5), of which 120 genes overlapped between the two time points.

**Figure 5:**
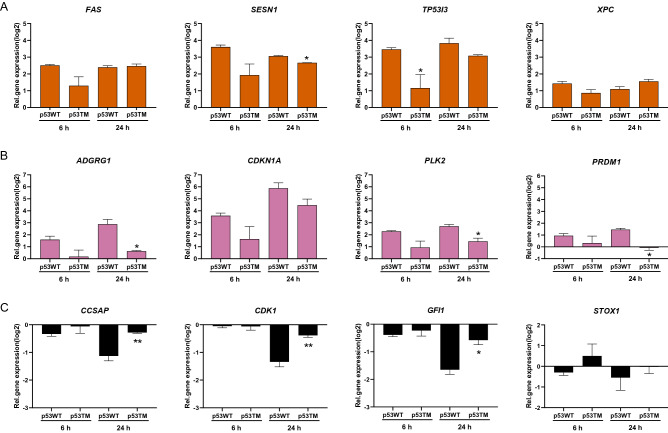
Confirmation of time-dependent impact of p53 TM on transactivation of target genes by quantitative Real-Time PCR. Selected genes whose transactivation were early (**A**) or continuously repressed (**B**), or downregulation repressed (**C**) are shown. Data show the mean ± SE (*n* = 3) normalized to the activity of GFP expressing cells. *P < 0.05, **P < 0.01 by one-way ANOVA compared to p53 WT.

TM of p53 reduced the expression level of 122 and 123 of these genes at 6 and 24 h, respectively (*P* < 0.05, 97.6% and 64% of the induced genes). In agreement with the low level of TM, presence of active AGT did not repress the transactivation of any genes at the two time points (not shown). To determine any time-dependent effects of TM on p53-dependent transactivation three clusters were identified; *early repression* (52 genes which were reduced only at 6 h), *continuous repression* (70 genes which were reduced at both time points) and *late repression* (53 genes which were reduced only at 24 h) of transactivation due to TM (Fig. 4B, Supplementary Tables S6–S8). Among the early repressed genes e.g. *FAS*, *SESN1, TP53I3,* and *XPC* were found, while the continuously repressed included *ADGRG1*, *CDKN1A*, *PLK2,* and *PRDM1* and genes with a late repression *ATF3*, *BMP7*, *CAV1* and *VDR* (Fig. 4C). Notably, the majority of the genes belonging to the latter cluster were not induced by expression of wild type p53 at 6 h. The effect of p53 TM on the transactivation was confirmed by quantitative real-time PCR for a selected number of early and continuously repressed genes (Fig. 5A-B). A comparison between the impact of p53 TM on transactivation of HCGs in the three groups and the transactivation potential of their response elements did not show any clear associations, suggesting that the observed temporal clusters are not related to response element functionality (Supplementary Fig. S1).

To further elucidate the impact of this temporal dynamics on fundamental cellular processes, gene ontology (GO) term enrichment analysis of Biological Processes between the three clusters was performed (Supplementary Table S9). Although the three clusters all contained unique genes, a clear overlap of processes was found between the genes whose transactivation were repressed early and continuously (Fig. 4D). These processes included GO terms related to DNA damage response (*Response to UV*, *Response to abiotic stimulus*, *Cellular response to DNA damage stimulus*, *Signal transduction in response to DNA damage*) and regulation of cell cycle (*Negative regulation of cell cycle*, *Cell cycle checkpoint*). As expected, genes annotated as being involved in p53 signalling (GO term: *Signal transduction by p53 class mediator*) were represented in all three clusters as was GO terms related to apoptotic signalling (*Intrinsic apoptotic signalling pathway, Apoptotic signalling pathway*). The enriched Biological Process GO terms associated with genes with a late repression of transactivation were more distinct compared to the two other clusters and included *Gland morphogenesis*, *Regulation of peptidase activity* and *Apoptotic processes involved in development*. These results suggest that TM of p53 can deregulate several canonical pathways which are important for cellular homeostasis and in a time-dependent manner.

### TM also affects transcriptional downregulation by p53

**Figure 6:**
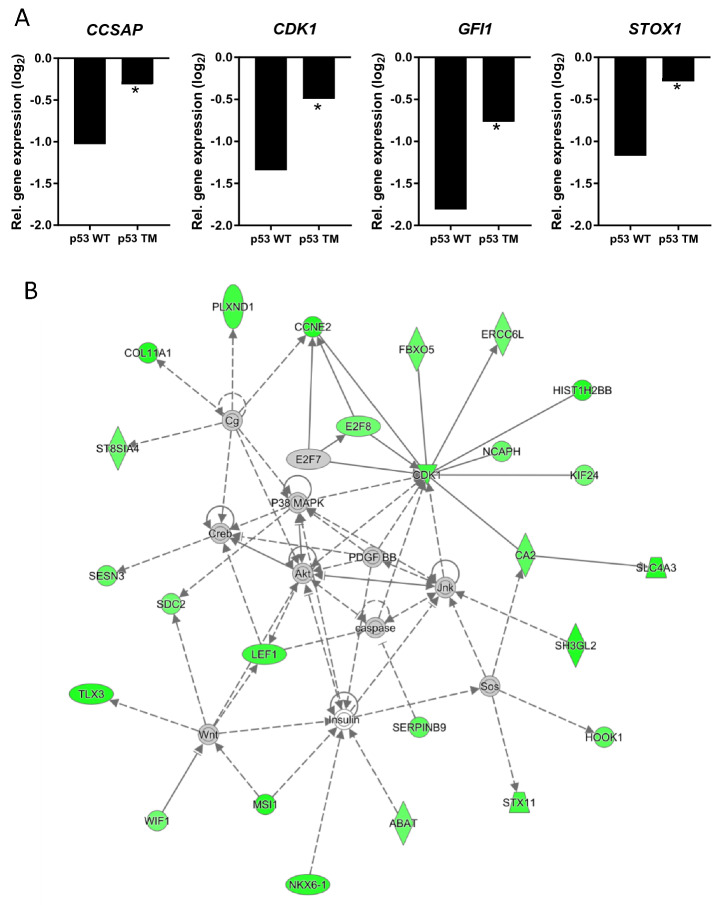
TM represses transcriptional downregulation by p53. (**A**) Examples of genes whose downregulation were deregulated as a result of p53 TM with *P < 0.05. (**B**) Network containing genes whose p53-mediated downregulation were repressed by TM at 24 h (in green). Grey nodes are upstream regulators or downstream effectors. Intact arrows indicate direct interactions, dashed arrows indirect relationships. The network was identified by IPA as the network with the highest score based on the 171 downregulated genes which were deregulated by TM.

As stated above, 9 and 383 genes were downregulated in response to expression of wild type p53 in H1299 cells at 6 h and 24 h, respectively. GO term enrichment analysis of the latter group revealed that many of these genes were associated with cell cycle regulation (Supplementary Table S10), confirming a functional p53-dependent downregulation of cell cycle genes in H1299 cells. TM of p53 repressed the downregulation of 171 genes at the late time point including *CCSAP*, *CDK1*, *GFI1*, and *STOX1* (*P* < 0.05, Fig. 6A, Supplementary Table S11), but no GO term enrichments could be found. The repressed downregulation of these genes was confirmed by quantitative Real-Time PCR (Fig. 5C). At the early time point, the corresponding number was 3 genes, two ncRNAs and the small GTPase protein *RASL11B* (*P* < 0.05, Supplementary Table S11). The 171 deregulated genes were further studied by pathway analysis using IPA. The analysis revealed that the E2F transcription factor family (i.e. E2F4, E2F7 and E2F8) was among the most important upstream regulators of these genes, including the cell cycle genes *CCNE2*, *CDK1* and *E2F8*. These genes were also all part of the top score network identified by IPA based on the 171 deregulated genes (Fig. 6B). The network contained genes annotated to be involved in Biological Processes *Cell differentiation* (e.g. *CA2*, *HOOK1* and *NKX6-1*), *Cell division* (e.g. *ERCC6L*, *FBXO5* and *LEF1*), *Response to stress* (e.g. *ABAT*, *SERPINB9* and *SESN3*) and *Homeostatic process* (e.g. *ABAT*, *CA2* and *SLC4A3*). The network also included members of MAPK, Akt, and Wnt protein families as upstream regulators and/or downstream effectors, which all have central roles in mediating cellular signalling in relation to differentiation and proliferation. The fact that TM also impacts on the indirect transcriptional downregulation by p53 underscores the potential role of TM in deregulating cellular mechanisms for homeostasis maintenance.

## Discussion

We have recently shown that expression of mutant R248W p53 due to TM resulted in repressed transactivation of p53 target genes with downstream impairment of G1/S cell-cycle checkpoint and activation of apoptosis in H1299 cells^10^. Here, we assessed the impact of TM on the transactivation activity of p53 genome wide by RNA-seq. The results revealed that TM of p53 repressed the transactivation of a large number of HCGs representing many canonical p53 tumour suppressor pathways. These effects were moreover found to display temporal patterns and to include deregulation of transcriptional downregulation by p53.

Critical to the tumour-suppressive function of p53 is its ability to bind sequence-specific DNA sites and transactivate expression of target genes, thus making p53 a susceptible target for mutations. The majority of p53 mutations, such as R248W used in this study, occur in the core DNA-binding domain and results in a reduced DNA-binding affinity^18,23^. Moreover, mutant p53 have been shown to exert dominant-negative effects over wild type p53 if both forms co-exist as they retain the ability to form tetramers but interfere with DNA-binding ability thus decreasing level of functional p53. The p53 dimers are formed co-translationally resulting in either mutant or wild type dimers, whereas tetramers are formed post-translationally by dimerization of existing dimmers^24^. Assuming that this model also applies to TM, the reported uridine misincorporation induced by *O*^6^-meG (with inactive DNA repair) in 47% and 18% of transcripts at 6 h and 24 h, respectively, would correspond to 72% and 33% of the expressed p53 protein being inactivated due to TM. These numbers are supported by the observed reduced DNA binding activity data at 20% and 80% activity, respectively, and the CIBERSORTx based estimation of cell population phenotype, which suggested that 65% and 48% of cells expressed a mutated p53 gene expression program. In all, based on misincorporation frequency, DNA binding activity data, and functional genomics analysis of gene expression profiles, our work demonstrates that TM of p53 has the potential to significantly alter its gene expression program and impact on cellular phenotype.

p53 primarily functions as a transcription factor regulating expression of numerous target genes and initiating a variety of cellular responses. Different cellular stresses, such as DNA damage, oncogene activation, ribosomal stress or hypoxia, are known to induce stabilization and activation of p53^25^. Mutagenesis can thus have a severe impact on several crucial cellular processes. The model of how p53 is mediating the regulation of gene expression in response to various stresses is highly complex and remains not well characterized^26^. Intensive studies for several decades have identified thousands of potential p53 target genes, however, recent meta-analyses have reported that most of the potential p53 target genes have a high likelihood of being false positives which are indirectly activated downstream the p53 signalling pathway, thus limiting the number of p53 HCGs to not exceed a few hundred^14^. Here, expression of wild type p53 induced the expression of 197 HCGs and the presence of TM repressed the transactivation of a majority of these genes, in a time-dependent manner. In agreement, different kinetics in expression profiles of target genes following p53 induction has been observed in vivo where different genes clustered in groups based on their function showed early, continuous or late response^15,16^. Cell cycle genes were among the early responders which support our findings that these genes also belonged to the cluster which was repressed by TM at the early time point. The majority of the HCGs identified as being deregulated by p53 TM in our previous study, including *APAF1*, *BBC3*, *BTG2*, *CDKN1A*, *PLK2* and *SFN*^10^, were here confirmed as such and belonged to the group of genes whose transactivation was continuously repressed. It was only *PMAIP1*, which encodes the pro-apoptotic NOXA protein, which belonged to the group of late repressed genes. The temporal regulation of p53 target genes remains poorly understood, however, various factors, such as highly diverse core promoters with different levels of preloaded RNA pol II, sequence variations and differences in binding affinities of p53 to response elements, p53 regulatory proteins and post-translational modifications and chromatin context, have been associated with the time-dependent activation of expression^16^. To our knowledge, the impact of genomic p53 mutagenesis on the temporal regulation of target genes has not been studied. Our results suggest that the observed temporal effects are not related to response element functionality, and that other factors likely determine the dynamics of TM-mediated deregulation of p53 transactivation.

TM of p53 also caused a deregulation of transcriptional downregulation by p53. Although the current model describes that p53 is solely a direct transcriptional activator, it indirectly exerts transcriptional downregulation through *CDKN1A*/p21 and other downstream effectors resulting in activation of cell cycle arrest^27^. Following transactivation, p21 represses cell cycle progression by binding CDK/cyclin complexes that are crucial for inactivation of retinoblastoma protein and release of E2F transcription factors. As a result, E2F4 repressor protein is recruited to cell cycle gene promoters which together with other proteins form the multiprotein repressor complex DREAM^17,28^. The key role of E2F transcription factors in the downregulation by p53 was also observed here. For example, the downregulation of G1/S cell cycle gene *CDK1,* which is mediated by p53 activation via p21 and E2F/DREAM proteins^27^, was deregulated by p53 TM.

Several studies have shown that dominant-negative p53 mutants, such as the R248W p53, are capable of altering gene expression patterns not simply due to loss of function of wild type p53 but also due to gain of new functions, thus an oncogenic gain of function phenotype has been proposed as a consequence of p53 mutagenesis^19,29^. However, evidence for additional oncogenic properties of mutant p53 remains inconsistent. Recent genome-wide approaches showed no evidence for gain of function phenotype of dominant-negative p53 mutants in patient-derived lymphocytes^30^. This is further supported by the data presented here where no alterations in gene expression profiles was observed in response to R248W p53 expression compared to the GFP control or wild type p53 at both early and late time-points.

Several DNA repair mechanisms operate in cells to maintain their genomic stability and survival. Even though *O*^6^-meG is not the most abundant DNA lesion, it is a major mutagenic lesion during both transcription and replication, causing base mispairing and thus leading to uridine misincorporation or GC → AT transition mutations, respectively^10,31^. The *O*^6^-meG lesion is directly eliminated from DNA in a single-step suicidal reaction by AGT, and AGT homologues have been identified in almost all studied organisms^32,33^. AGT is highly efficient in suppressing point mutations and genotoxicity induced by *O*^6^-meG in vitro and in vivo and the most important defence against its associated tumorigenesis^34^. We reported here that uridine misincorporation induced by *O*^6^-meG in H1299 cells with inactive AGT was detected in 47% of p53 transcripts at 6 h which decreased to 18% at 24 h, the latter being in agreement with our previous study^10^. We recently showed that presence of *O*^6^-bzG inhibited the AGT activity > 95% for up to 24 h post transfection^10^. The observed time-dependent decrease of uridine misincorporation might thus indicate that other repair systems could play a backup role for AGT. This is supported by a lowered but maintained ability to repair *O*^6^-meG in *Mgmt*^–/–^ mice^35^. A possible overlap in the repair of *O*^6^-alkylated lesions by the human AlkB homologue 2 (ABH2) protein and base excision repair pathway has been suggested, but not yet established^34^. Studies have also shown that both *E. coli* and human excinucleases belonging to the nucleotide excision repair (NER) pathway were able to remove *O*^6^-meG in vitro, however with low efficiencies^36^. Alkyltransferase-like proteins (ATLs), thus far only identified in prokaryotes and lower eukaryotes, can trigger the NER pathway by binding alkylated DNA^37,38^. However, strongest evidence exists for the human MutH homologue 1 (MTH1) protein^39^, which belongs to a protein family that is responsible for removal of oxidized NTPs from the nucleotide pool^40^. Jemth et al. recently showed that MTH1 efficiently catalysed the hydrolysis of *O*^6^-methyl-dGTP and that MTH1 deficiency sensitized human cells to the alkylating drug Temozolomide^39^. This suggests that MTH1 could contribute to the observed repair of *O*^6^-meG in cells with inactive AGT.

Several lines of evidence have shown that TM can be a DNA replication-independent source of mutated proteins with altered functions. It has previously been suggested that TM could be a contributing mechanism to anomalies during development, especially neurodevelopmental deficits, where exposure to DNA damaging agents could have devastating effects on transcriptional outcome of genes required for normal development and function due to TM^41,42^. Here we show that TM of p53 resulted in deregulated transactivation and downregulation of many target genes involved in regulation of cellular processes, such as cell-cycle arrest, apoptosis, and DNA damage response, all of which are crucial for its tumour suppressor function. This means that even though TM occurs in transcripts from only one gene, this can activate aberrant gene expression programs that significantly change the cellular phenotype. Cancer development is driven by an expansion of mutant subclones within a neoplasm which is dependent on a selective pressure exerted by internal or external factors^43^. Our and other’s data^10,12^ thus suggest that even if TM only affects a few cells, this could be enough to change phenotypic traits of already initiated pre-neoplastic cells which allows for clonal expansion and further development of cancer.

## Materials and methods

### Construction of vectors

To study impact of TM on p53 function as a transcription factor, vectors pGFP, pP53^WT^, pP53^RW^ and pP53^O6-meG^ were constructed. Detailed description of construction of the vectors, which was based on the vectors used for studying the effects of *O*^6^-meG on transcription^9^, can be found in our previous publication^10^. The vectors used in this study contained two genes, *TP53* and *GFP*, controlled by two independent tetracycline response element-tight (TRE-tight) promoters and an F1 origin of replication for production of ssDNA that corresponds to the coding strand of the two genes. These plasmids contained no known mammalian origin of replication, thereby limiting effects of replication^9^.

Each vector was named according to the sequence it contained; pGFP contained only GFP coding sequence, pP53^WT^ contained the wild type p53 coding sequence, whereas pP53^RW^ contained the p53 coding sequence with a single base change at codon 248 (CGG → TGG) resulting in the expression of mutant R248W p53. Vector pP53^O6-meG^ contained the wild type p53 coding sequence with an *O*^6^-meG on the template strand opposite the first C of the arginine codon CGG at codon 248. During transcription C or U could be inserted across the lesion into nascent transcripts and resulting in either CGG codon coding for arginine and expression of wild type p53 or UGG codon coding for tryptophan and the mutant RW p53. Generation of pP53^O6-meG^ vector with site specific base modification involved production of wild type p53 containing ssDNA, annealing and ligation of a site-specifically *O*^6^-meG-modified 11-mer oligo (Sigma-Aldrich, Stockholm, Sweden) to a gapped duplex construct, and purification of closed circular dsDNA, as described previously^10^.

### Cell culture and transfection

The human p53-null lung carcinoma H1299 cell line (CLR-5803, ATCC, Middlesex, UK) was cultured in DMEM (Gibco by Life Technologies, Stockholm, Sweden) supplemented with 10% FBS, 1 mM L-glutamine, 100 U/ml penicillin and 100 µg/ml streptomycin in a humidified 5% CO_2_ incubator at 37 °C. These cells, which are p53-null for both alleles and a common model for studying the effects of p53 mutagenesis, were used to avoid any interfering effects from endogenous p53. Cells between passages 7 and 12 were transiently transfected with the vectors using Lipofectamine 2000 (Invitrogen, Stockholm, Sweden) in ratio 1:2 DNA:Lipofectamine 2000 following manufacturer’s protocol. In order to block repair of *O*^6^-meG by AGT, 10 µM *O*^6^-bzG (in DMSO, Santa Cruz Biotechnology, Heidelberg, Germany) was added to the cells 1 h before transfection. We have previously shown that this cause a > 95% inhibition of AGT’s DNA repair activity^10^. Cells were harvested 6 and 24 h after transfection. Based on FACS analysis of the co-expressed GFP, transfection efficiencies of the different vectors were 7–12% at 6 h and 30–50% at 24 h after transfection.

### Western blotting

Cell lysates were obtained in lysis buffer containing Halt Protease & Phosphatase Single-Use Inhibitor Cocktail (Thermo Scientific), 20 µg of protein were loaded on 12% polyacrylamide gel and then transferred on nitrocellulose membrane. The membrane was blocked with Odyssey blocking solution (LI-COR, Lincoln, Nebraska) at room temperature for 1 h and followed by an incubation overnight with primary antibodies p53 DO-1 mouse monoclonal IgG (1:5,000, Santa Cruz Biotechnology) and Cdk2 rabbit polyclonal IgG (1:10,000, Santa Cruz Biotechnology) at 4 °C. The membrane was then incubated with secondary antibodies Goat anti-mouse IgG (1:5,000, IRDye® 680RD, LI-COR) and Goat anti-rabbit (1:10,000, IRDye® 800CW, LI-COR) at room temperature for 1 h in dark. Odyssey detection system (CLx, LI-COR) was used to visualize the immunoreactive bands. Image J software was used to quantify the density of the bands.

### Transcription factor assay

Nuclear proteins were extracted from approximately 1.5 × 10^6^ cells following transfection using a Nuclear Extract kit (Active Motif, La Hulpe, Belgium) according to the manufacturer’s instructions. Protein concentrations were determined by using Qubit 4 fluorometer (Qubit protein assay kit, Invitrogen). Transcription factor activity of p53 in 5 µg of nuclear extracts was determined using a p53 ELISA-based transcription factor assay (TransAM assay, Active Motif) according to the manufacturer’s protocol. The nuclear extract was incubated in a 96-well plate immobilized with an oligonucleotide containing a p53 consensus binding site (5′–GGACATGCCCGGGCATGTCC-3′). This oligonucleotide is specifically bound by p53 in the nuclear extract, upon DNA binding an accessible epitope of p53 is recognized by the primary antibody, followed by a secondary HRP-conjugated antibody. Nuclear extract from MCF7 cells (H_2_O_2_ treated) was included as a positive control. The colorimetric readout at 450 nm was determined in a microplate reader (Infinite F200 Pro, TECAN, Switzerland). Differences in DNA binding activity were analysed with one-way ANOVA (*P* < 0.05) using GraphPad Prism 7.0 software.

### Cell sorting and RNA purification

Sorting of transfected cells was based on the fluorescence signal of the GFP reporter gene using BD FACSAria III or BD FACSAria Fusion. Total RNA from sorted cells was purified using the RNeasy Mini Kit (Qiagen, Aarhus, Denmark) according to the manufacturer’s protocol. Quality of RNA was confirmed by Agilent 2100 Bioanalyzer (Agilent Technologies, Stockholm, Sweden).

### Quantitative Real-Time PCR

cDNA was produced by following the instructions of high capacity cDNA reverse transcription kits (Applied Biosystems, Stockholm, Sweden). Analysis of gene expression was performed in duplicates using Maxima SYBR green qPCR master mix (Thermo Fisher Scientific, Stockholm, Sweden) with detection on a QuantStudio 5 Real-Time PCR System (Applied Biosystems). Three-step thermal cycling was performed with initial denaturation at 95 °C for 10 min, followed by 40 cycles of 95 °C for 15 s, 60 °C for 30 s, 72 °C for 30 s followed by melt curve analysis. Comparative Ct (∆∆Ct) analysis was employed to estimate relative mRNA quantification with 60S acidic ribosomal protein P0 (*RPLP0*) as reference gene. KiCqStart primers obtained from Sigma Aldrich (Stockholm, Sweden) are presented in Supplementary Table S1. Differences in expression levels were analysed with one-way ANOVA (*P* < 0.05) using GraphPad Prism 7.0 software.

### RNA-seq

RNA libraries from biological triplicates were prepared using TruSeq Stranded Total RNA Library Prep Kit with Ribo-Zero Gold (Illumina, Stockholm, Sweden) according to the manufacturer’s protocol. Briefly, high-quality total RNA (0.5–1 µg) was subjected to rRNA depletion. Purified remaining RNA with RNAClean XP beads (Beckman Coulter, Stockholm, Sweden) was fragmented, primed with random hexamers and reverse-transcribed using SuperScript III (Invitrogen, Stockholm, Sweden) to generate cDNA. Then dscDNA was generated and purified using MagSi-NGS^PREP^ Plus beads (MagnaMedics, Stockholm, Sweden). Adenylated dscDNA was indexed using provided RNA-seq Adapter Indexes (Supplementary Table S2) and subsequently amplified for 15 PCR cycles. Prepared libraries were validated before sequencing using Qubit for quantification (Invitrogen, Stockholm, Sweden) and Agilent 2100 Bioanalyzer for size distribution. A pool of 10 libraries were sequenced on an Illumina NextSeq 500 instrument using NextSeq 500/550 High Output v2 kit (75 cycles) and the single-read sequencing protocol.

### Sequencing data analysis

The RNA-seq reads were aligned to human reference genome hg38 with STAR v2.5.3a^44^ using default setting, except maximum intron size as 1 Mb. The counts of reads mapped to genome annotations in GENCODE v27 were calculated using featureCounts program in Subread v1.5.2^45^. The gene counts were further imported to DESeq2 v1.20^46^ for differential expression analysis using Wald significance tests with interaction of time and vector condition. The results have been deposited in the NCBI GEO (accession number GSE138853).

### Quantification of TM

The frequency of TM at codon 248 of p53 and the position corresponding to the site-specific *O*^6^-meG in the sequencing data was determined using bcftools v1.8^47^. Additionally, frequency of TM at 6 h after transfection was determined by tetra-primer ARMS-PCR followed by sequencing^11,48^. In brief, colony PCR was performed on 100 clones (from four replicates) using the primers as indicated in Supplementary Table S3; the PCR products resolved on a 3% agarose gel; and colonies that amplified with the mutant-specific primers propagated for plasmid isolation and subsequent confirmation by sequencing. Differences in levels of TM were analysed with one-way ANOVA (*P* < 0.05) using GraphPad Prism 7.0 software.

### Functional genomics analysis

Analysis was performed on differentially expressed genes (DEGs, 3,166 ID mapped genes with fold change > twofold and *P* < 0.05) and direct effects on p53 target genes were further determined by comparison with a list of high-confidence p53 target genes (HCGs) recently identified by meta-analysis of 16 genome-wide data sets and 319 individual gene studies^14^. Gene ontology (GO) term and pathway enrichment analysis was performed using ToppCluster (www.toppcluster.cchmc.org)^49^, GOrilla (www.cbl-gorilla.cs.technicon.ac.il)^50^ and Ingenuity Pathway Analysis (IPA, Qiagen, Aarhus, Denmark). Cut-off for GO Biological Process term enrichment was set to *P* < 0.05 using FDR adjustment. GO terms were summarized using REVIGO (https://revigo.irb.hr/)^51^. Correlation between impact of p53 TM on transactivation of HCGs and the transactivation potential of their response elements was analysed using a grading scale compiled by the R package p53retriever and using the highest grade for each HCG^52^. CIBERSORTx (https://cibersortx.stanford.edu/)^53^ was used to estimate the percentage of cells displaying a mutant p53 phenotype after TM induction. Bulk RNA-Seq data was used to estimate the fraction of cells displaying a mutant p53 cellular phenotype (using wild type and mutant R248W as references). DEGs from the two time points were analysed using recommended default job parameters.

## Supplementary information

Supplementary Figure S1.

Supplementary Tables.

## Data Availability

The RNA-seq data have been deposited in the NCBI GEO (Accession Number GSE138853).
