# Supplementary material for: Transcriptional mutagenesis dramatically alters genome-wide p53 transactivation landscape
Source: Sci Rep. 2020 Aug 11;10:13513. doi: 10.1038/s41598-020-70412-4 (PMC7419513; doi:10.1038/s41598-020-70412-4)
Supplement: Supplementary file 1 — Supplementary Figure S1. [file 41598_2020_70412_MOESM1_ESM.docx]

**Transcriptional mutagenesis dramatically alters genome-wide p53 transactivation landscape**

Shuo Liang^1‡^, Monika Ezerskyte^1‡^*, Jingwen Wang^2^, Vicent Pelechano^2^, Kristian Dreij^1^*

1. Unit of Biochemical Toxicology, Institute of Environmental Medicine, Karolinska Institutet, 171 77 Stockholm, Sweden
2. Science for Life Laboratory, Department of Microbiology, Tumor and Cell Biology, Karolinska Institutet, 171 77 Stockholm, Sweden

^‡^These authors contributed equally.

*Corresponding authors:

Monika Ezerskyte; monika.ezerskyte@ki.se

Kristian Dreij; kristian.dreij@ki.se

Short title: Gene expression changes in response to p53 TM

Keywords: transcriptional mutagenesis, tumour suppressor p53, differential expressed genes, RNA-seq

**Figure S1.** Comparison of response element (RE) functionality for the TM-deregulated genes in the different temporal groups shown in Figure 4. Analysis was performed using the R package p53retriever and using the highest functionality grade for each gene.
